# Editorial: Manual on how to make and use an organoid: current and future directions

**DOI:** 10.3389/fendo.2023.1306505

**Published:** 2023-10-19

**Authors:** Sarah A. Hannou, Akshay Bareja

**Affiliations:** ^1^ Department of Endocrinology, Baylor College of Medicine, Houston, TX, United States; ^2^ Department of Medicine, Duke University School of Medicine, Durham, NC, United States; ^3^ Duke Molecular Physiology Institute, Duke University School of Medicine, Durham, NC, United States

**Keywords:** organ in dish, tissue-engineered-cell based *in vitro* models, personalized medicine, endocrinopathies, pituitary disorders, endometrial diseases

Decades of research have shown that conventional two-dimensional cell line-based *in vitro* models do not fully recapitulate the complexity of tissue composition or disease development. On the other hand, animal models, due to species-specific differences, do not accurately reflect human pathophysiology. They also typically fail to mirror the genetic complexity and variation associated with various human diseases.

Recent advances in stem cell and developmental biology have led to the development of an *in vitro* model system known as “organoids”. Organoid culture consists of stem cell-derived three-dimensional cellular units with heterogeneous cellular composition capable of reproducing the biological structure and function of their organ of origin. Organoid cultures are genetically stable, can be indefinitely propagated and frozen, thereby providing ease of use, storage, and transfer which make it an excellent tool for disease modeling, drug discovery, and regenerative medicine.

Recent breakthroughs in the field have enabled a better understanding of the functionality, pathogenicity, and targeted treatment of complex diseases such as cardiometabolic disease and endocrine disorders; however, despite rapid progress, there are still inherent bioengineering limitations. The use of key reagents such as scaffolding matrices or growth factors are still a matter of debate. Additionally, various labs have each developed their own protocols for generating organoids to model endocrine biology and disorders, and there isn’t yet consensus between these groups. In this Research Topic, we therefore aimed to create a collection of original research articles and clinical studies that discuss the use of organoids as a systems approach to study complex endocrine diseases and endocrinopathies. We aimed to create a resource with accurate and well-defined protocols for endocrine organoid culture that follow standardized procedures that are easily reproducible. Articles in this Research Topic accurately describe protocols to grow healthy and patient-derived organoids that can be used for transplantation or to evaluate possible treatment strategies, showing the therapeutical potential of using organoids. The current Research Topic of articles has brought to light the importance of using this new strategy to study, treat and prevent the development of endocrine disorders. While these articles focus on pituitary and endometrial disorders, they exemplify an approach that can be applied to a wide range of endocrinopathies.

In both studies submitted by Kano et al. and Sasaki et al. the authors focused on implementing strategies to combat the effects of pituitary disorders. As a central part of the endocrine system, the pituitary gland produces several hormones and controls other glands, such as the thyroid gland and the adrenal glands. Conventional treatment consists of replacement therapy which is hard to adjust and has limited benefits. The authors showed that these organoids can be derived either from pluripotent or tissue-derived stem cells. Kano et al. described a protocol for generating hypothalamus-pituitary organoids from human pluripotent stem cells (hPSCs) that can potentially be used as engraftment organs. This strategy was successfully implemented by Sasaki et al., where the authors showed that human embryonic stem cells (hESC)-derived pituitary organoids can be successfully engrafted into and function in the subcutaneous tissue of hypopituitary SCID mice thereby rescuing pituitary function.

Diseases impacting females’ reproductive organs such as infertility, endometrioses or gynecologic malignancies pose a critical health concern. The need to establish *in vitro* models to study endometrial cells is crucial to understanding the mechanism that contributes to normal endometrial function and the origins of related diseases. Katcher et al. highlighted in their work the importance of increasing patient specimens’ diversity with respect to race, ethnicity and histologic subtype of the primary cancer tissue. The authors developed a living biobank by collecting samples and growing organoids from both endometrial tumor tissue and matched normal endometrium. These organoids were than passaged long term, banked, and can be utilized for downstream histological and genomic characterization as well as functional assays in response to therapeutic drugs. The same strategy was used by Zhou et al. where the authors used a more global proteomics approach to screen endometrial organoids derived from endometrial biopsies from women with primary infertility and normal fertility. This model allowed the authors to identify specific factors that regulate blastocyst adhesion and implantation offering great hope for the development of treatment for infertility. With a less invasive method for collecting endometrium specimens and taking advantage of women’s natural reproductive cycle and endometrium desquamation, Hewitt et al., implemented a method for the isolation of paired endometrial epithelial organoids and stromal cells from menstrual fluid collected from individual women. This approach allowed the authors to isolate both epithelial and stromal cells from a single menstrual fluid sample, allowing them to study organoids and cells in a paired manner. This protocol can greatly enhance our understanding of the role of epithelial and stromal cells alone and in coordination.

As outlined by articles in this Research Topic, organoid models hold great promise in improving human health. They can be dissected and interrogated for use in fundamental mechanistic studies, and can be used in diagnostics, disease modelling, drug discovery and personalized medicine. While there is a great enthusiasm for and interest in this technology, more work is needed to overcome currents limitations.

## Author contributions

SH: Conceptualization, Writing – original draft, Writing – review & editing. AB: Writing – original draft, Writing – review & editing.

